# Decreasing of Leaching and Improvement of Geopolymer Properties by Addition of Aluminum Calcium Cements and Titanium Oxide

**DOI:** 10.3390/ma13030495

**Published:** 2020-01-21

**Authors:** Michał Łach, Kinga Korniejenko, Janusz Walter, Anna Stefańska, Janusz Mikuła

**Affiliations:** Institute of Materials Engineering, Faculty of Materials Engineering and Physics, Cracow University of Technology, Jana Pawła II 37, 31-864 Cracow, Poland; kkorniej@gmail.com (K.K.); janusz.walter@mech.pk.edu.pl (J.W.); m-2@mech.pk.edu.pl (A.S.); jamikula@pk.edu.pl (J.M.)

**Keywords:** geopolymers, leaching ions, aluminum calcium cements, titanium oxide

## Abstract

The article presents the latest results of the leaching of alkali from geopolymers depending on the introduced additions in the form of aluminum calcium cement and nanometric titanium oxide. Aluminum calcium cements were introduced in two variants: G40 (>40% Al_2_O_3_) and G70 (>70% Al_2_O_3_) in amounts of 0%, 2%, and 4% by weight. Titanium oxide was also incorporated in amounts of 2% and 4% by weight. The results of conductivity tests of solutions in which geopolymers were immersed were carried out. On this basis, it was found that geopolymers cured in the aquatic environment have a lower risk of efflorescence in the later periods of their use due to leaching of compounds at the stage of aquatic curing. In addition, it was found that the addition of calcium aluminum cements decreases the leaching of substances from geopolymers. It was also found that geopolymers based on an 8 M NaOH solution have greater leaching than when using a 10 M solution. The results of the compressive strength tests for the tested samples were also presented.

## 1. Introduction

Due to the EU (European Union) policy on reducing greenhouse gas emissions, the important driving force for the implementation of geopolymer concrete is the prospect of significantly reducing CO_2_ emissions in the production process compared to CO_2_ emissions in the production of Portland cement [1,2,3]. On a global scale, the cement industry is responsible for approximately 5% of global industrial CO_2_ emissions [4,5]. The key barriers associated with the widespread use of geopolymers are problems with the so-called precursors. In the case of metakaolin, it is the high price of this raw material, while in the case of fly ash, it is the large variability of its oxide composition and morphology [5]. Fly ash is a very diverse material, the composition of which depends not only on the impurities present in coal before combustion but also on the combustion process and subsequent flue gas cleaning process.

A very important barrier related to the use of geopolymer products is the occurrence of so-called efflorescence on geopolymer surfaces, regardless of whether they are based on metakaolin, fly ash, volcanic tuff, or other aluminosilicate precursors. This is not just an aesthetic problem. The formation of efflorescence can also cause degradation of the geopolymer structure. Currently, many scientific centers are working on limiting the formation of efflorescence and developing additives to prevent their formation [6,7,8].

The formation of efflorescence is associated with the leaching of geopolymers of sodium or potassium ions [9]. If the products come into contact with moisture, water, due to capillary forces, they leach alkali ions and “transfers” them to the surface [1,6,10]. After evaporation of water, these ions react with CO_2_ in the atmosphere and form hydrated sodium or potassium carbonates, e.g., Na_2_CO_3_ ∙ nH_2_O, NaHCO_3_ ∙ nH_2_O, K_2_CO_3_. This happens especially when the material has an excess of alkaline substances and the material is characterized by high porosity [11]. This applies primarily to geopolymers with a high Na_2_O/Al_2_O_3_ ratio. Studies carried out to date on efflorescence on geopolymers have shown that white efflorescence on the surface of geopolymer samples is Na_2_CO_3_ · 7H_2_O [12]. The identified carbonate crystallizes from inside the pores and “grows” out of the sample. Research on the ^23^Na NMR MAS spectrum indicates that alkali metals are bound by Al in the structure of the Si-O-Al chain in the form Na,K(H_2_O)_n_^+^ and not in the form Na^+^ or K^+^. Alkali metal bonding in the form of Na,K(H_2_O)_n_^+^ complexes is weaker than direct Na^+^ bonding, which is the reason for the ease of leaching of alkali metals from alkali-activated structures and geopolymers.

The methods used to prevent efflorescence in the case of geopolymers are either ineffective or not completely effective [12]. The only effective method, based on annealing of geopolymers at temperatures above 600 °C [13], significantly affects the structure and properties of geopolymers. For most geopolymer products, this is undesirable and also increases the cost of manufacturing the product.

Controlling the activator SiO_2_/Na_2_O molar ratio and the total Na/Al molar ratio is a difficult issue that requires optimization of these parameters for virtually every fly ash supply, which is unacceptable in industrial conditions. This is due to the large variability of fly ash oxide composition from various supplies even within one power plant and one and the same power boiler. In addition, using this method requires a trade-off between geopolymer compressive strength and correspondingly low alkali scrubbing, which is achieved by reducing the total Na/Al molar ratio. [8,13]. Meanwhile, in some solutions, compressive strength is the basic structural parameter.

Special additives in the form of metakaolin or blast furnace slag are practically not tested. Significantly better effects are obtained by the addition of calcium aluminates, in particular in the form of CaAl_2_O_4_. The presence of additional Al leads to a higher degree of crosslinking in the binding gel, and the geopolymer binder structure additionally develops higher strength and stronger alkali binding, which reduces efflorescence [14]. However, this method is not entirely effective, and the optimal level of calcium precursor replacement is 6% to 8%. The main role of the base in the initial phase of the polymerization process is to ensure a sufficiently high pH to start the distribution of covalent bonds (Si-O-Si and Al-O-Si) of the glass phase of the aluminosilicate material and then to balance the growing aluminosilicate gel [7,14].

Alkalis remain in the (N,K)-A-S-H gel nanostructure as unnecessary carrier residues from the activating solution [8,15,16]. The (N,K)-A-S-H gel can be considered as randomly oriented Al and Si polymer chains providing wells of sufficient size for hydrated Na or K ions in the form of Na(H_2_O)_n_^+^ or K(H_2_O)_n_^+^ ions [7,14].

Na(H_2_O)_n_^+^ and K(H_2_O)_n_^+^ ions can be replaced in the geopolymer chain by other cations present in the system, such as H_3_O^+^, but also Al(OH)_2_^+^ or Al_n_(OH)_m_^(3n−m)+^, or cations Fe, Mg, and Ca, which can also offset the negative Al^IV^ charge. The hydronium ion or hydrated hydrogen cation, H_3_O^+^, is the simplest oxonium ion. In aqueous solutions, it is formed as a result of self-ionization of water, 2H_2_O↔H_3_O^+^+OH^−^, then it is further hydrated. In an aqueous solution, this group is solvated by water molecules, which causes further hydration to H_3_O^+^(H_2_O)_n_, where n depends on temperature, solutes, and pressure, forming, among others, the Zundel cation H_5_O_2_^+^ and the Eigen ion H_9_O_4_^+^. Cryometric measurements indicate that the hydrated ion has an average size corresponding to H_3_O^+^(H_2_O)_6_. The Eigen cation is more durable than both the hydronium ion and the Zundel cation. The Eigen cation is formed by joining three water particles to the H_3_O^+^ hydronium ion: H_3_O^+^+3H_2_O → H_9_O_4_^+^.

As the above analysis shows, in order to protect sodium geopolymers against efflorescence, some sodium cations should be replaced, e.g., with H_3_O^+^(H_2_O)_n_ hydrogen cations, Al(OH)_2_^+^ cations, and Ca cations, and mobile sodium cations simply removed from the geopolymer structure.

It is also possible to include additives limiting the capillary porosity of the structure.

The source of aluminum and calcium ions in this study were calcium aluminates introduced in the form of aluminum calcium cements.

TiO_2_ titanium oxide was an additive that changed the porous structure of the geopolymer; the porous structure of the geopolymer was also changed by hydrothermal treatment [17,18,19]. There are also studies confirming that improvements in geopolymer properties can be obtained through the addition of GGBS (Granulated Blast-Furnace Slag). Additionally, such arrangements contribute to the management of waste from the building industry [20,21].

The aim of the study was to investigate the effect of aluminum calcium cements and titanium nanoxide on strength properties and ion leaching from geopolymer matrix. The impact of geopolymer additives can have a significant effect on the leaching of substances, which also affects the efflorescence on geopolymer surfaces. The research also determined the impact of NaOH solution concentration on ion leaching from geopolymers. Mobile sodium cations were removed by curing the samples in a water bath with a properly selected frequency of water exchange over 28 days.

## 2. Materials and Methods

Fly ash from the Skawina CHP plant (Skawina, Poland) was used as the precursor. The oxide composition of the precursor determined by the XRF method is shown in Table 1.

The addition of calcium aluminates was introduced in the form of calcium aluminate cements, which were the source of aluminum and calcium ions (Table 2). Górkal 40 cement and Górkal 70 cement manufactured by Górkal Cement (Górka Cement Sp. Z o.o.; Trzebinia, Poland) were used.

Geopolymer samples of size 50 mm × 50 mm × 50 mm were prepared for research on the basis of fly ash with the addition of G40 and G70 alumina cement and with the addition of titanium oxide.

Calcium aluminum cements were introduced in an amount of 2%, 4%, 6%, and 8% by weight, while titanium dioxide was introduced in an amount of 2%, 4%, and 6% by weight. TiO_2_ was introduced in the form of nanometric titanium oxide (Commercially available titania Aeroxide P25 (Degussa). 80% anatase (d = 21 nm) and 20% rutile (d = 50 nm), via TiCl_4_—fumed gas synthesis, Degussa, Germany).

Geopolymers were prepared using 8 and 10 M aqueous NaOH along with aqueous sodium silicate (1:2 ratio). The geopolymer compositions also included construction sand in a 1:1 ratio (sand:fly ash).

To produce geopolymer masses, technical sodium hydroxide in the form of flakes and an aqueous solution of sodium silicate R-145 with a 2.5 molar module and a density of about 1.45 g/cm^3^ were used.

Table 3 shows the compositions of the prepared geopolymer compositions with additives.

After obtaining a homogeneous mass with a dense plastic consistency, the mixtures were transferred to molds, which were then subjected to vibration on a vibrating table. The formed geopolymer concretes were heated in a laboratory dryer for 24 h at 75 °C at atmospheric pressure. After 24 h, the samples were drawn and demolded.

Leaching ability tests were conducted by immersing 50 mm × 50 mm × 50 mm geopolymer samples completely in 250 cm^3^ of distilled water. Periodically, water was exchanged to lower the salt concentration in the solution and accelerate the diffusion of ions into the solution.

Measurements of conductivity solutions in which geopolymer samples were immersed were successively carried out. Measurements were made using the Zahner IM6e electrochemical station by measuring the conductivity of the solution in a specially prepared conductivity vessel with two identical electrodes, passing a current between the electrodes at a frequency of 50 kHz and an amplitude of 100 mV. The cell constant for conductivity measurements was determined using three standard solutions of potassium chloride (KCl) at concentrations of 0.01, 0.1, and 1 M, whose conductivity is known. Then, NaOH solutions with known concentrations were prepared, and the dependence of the conductivity of the solutions on the concentration at 25 °C was determined using a previously determined cell constant. Before each measurement, they were washed using ultrasound to remove any possible residues of previously tested solutions.

Conductivity measurements were taken of the solutions in which the geopolymer samples were immersed. An equation describing the dependence of the solution conductivity on the NaOH concentration was determined. Using this equation, the theoretical mass of eluted hydroxide was calculated assuming that only sodium hydroxide undergoes elution.

Compressive strength tests according to EN 12390-3, using Matest 3000 kN (Matest, Treviolo, Italy), were conducted on cubic samples 50 mm × 50 mm × 50 mm conditioned at room temperature for 28 days. Five repetitions were carried out for each type of sample.

## 3. Results

### 3.1. Results of Conductivity Testing

The results of conductivity testing of solutions formed after curing of geopolymer samples are presented below.

**Figure 1:**
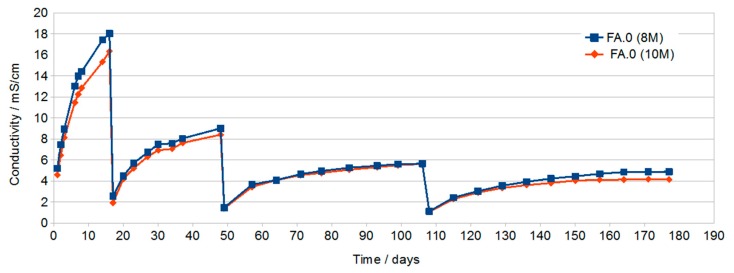
Of samples FA.0 (8 M) and FA.0 (10 M) as a function of immersion time with periodic water changes.

The conductivity of the samples shown in Figure 1 increases with increasing immersion time. During the first dozen or so days of testing, a sample prepared using the 8 M activator has a significantly higher conductivity, compared to a 10 M sample, which indicates a faster increase in the concentration of ions in the solution—probably larger pores or a much larger number of open pores. After a longer time and subsequent water changes, the conductivity of both samples obtains similar values. The final measurements made after about 4 months reach values of about 4 to 5 mS/cm with a clear stabilization of the value for the 10 M sample, which indicates the equilibrium between the concentration of eluted ions in the geopolymer and in the solution. This condition was not achieved in the 8 M sample.

The 8 M variant was chosen for further studies on the effect of the additives on leaching due to the higher conductivity values of the solution in the initial period.

**Figure 2:**
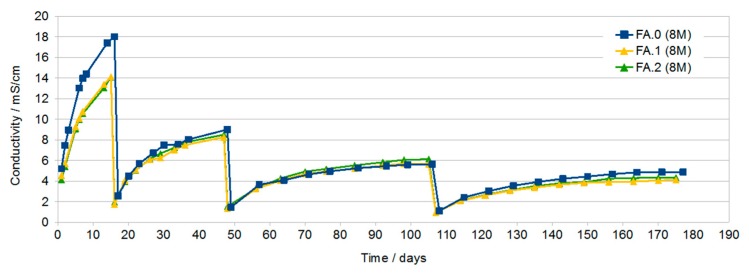
Of samples FA.0 (8 M), FA.1 (2% G40), and FA.2 (4% G40) as a function of immersion time with periodic water changes.

Figure 2 shows the changes in conductivity of samples produced with the participation of the 8 M activator with the addition of G40 in the amount of 2% and 4% (FA.1, FA.2, respectively) and sample FA.0 (8 M) without the addition of G40 as a reference sample. For the first water change, the effect of the G40 additive is clearly visible. Samples with the addition of G40 have about 4 mS/cm less conductivity after 15 days. With a further increase in time and after subsequent water exchanges, the conductivity of all samples approached about 5 mS/cm (after obtaining such conductivity, no blooms appear).

**Figure 3:**
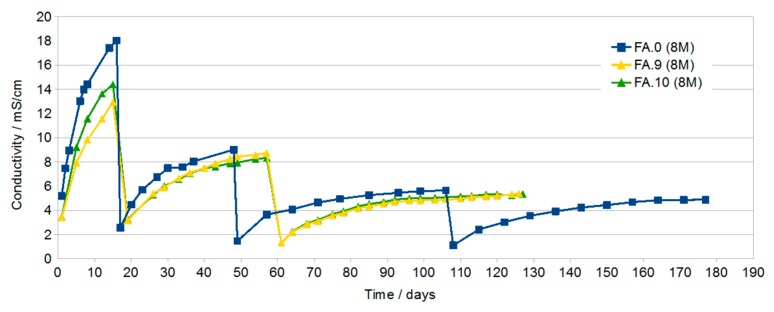
Of samples FA.0 (8 M), FA.9 (2% G70), and FA.10 (4% G70) as a function of immersion time with periodic water changes.

Figure 3 presents changes in conductivity of samples produced with the participation of the 8 M activator with the addition of G70 in the amount of 2% and 4% (FA.9 and FA.10, respectively) and sample FA.0 (8 M) without the addition of G70 as the reference sample. The course of the curves is very similar to those shown in Figure 2.

**Figure 4:**
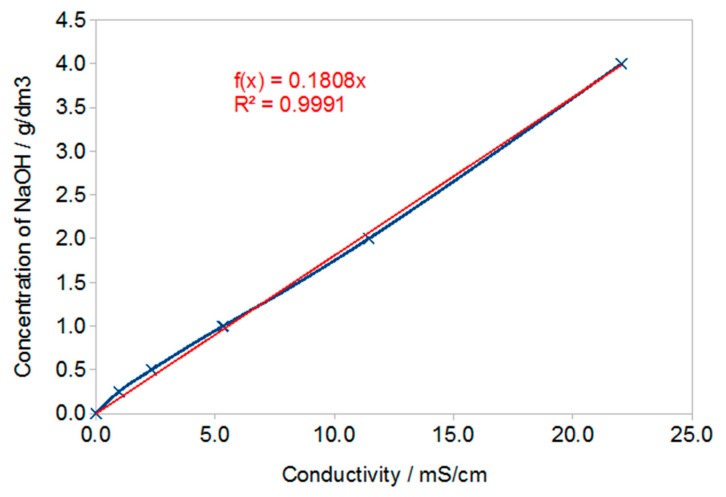
Dependence on NaOH concentration.

**Figure 5:**
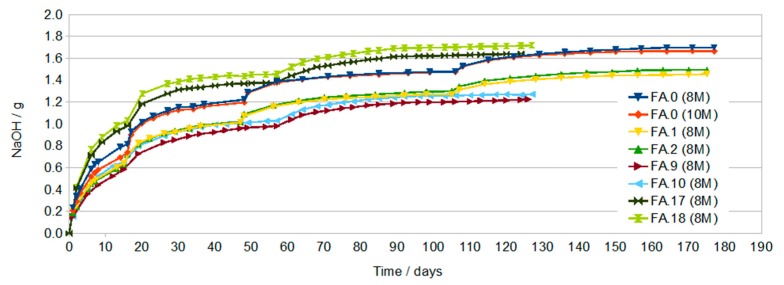
Of NaOH washed out (in 250 cm^3^ of solution).

Using the dependence shown in Figure 4, the hydroxide concentration in the solutions was calculated, and then taking into account the volume of solutions, the amounts of NaOH washed out in grams after subsequent washes were calculated. The NaOH content in subsequent solutions was summed for each of the samples and presented in the graph (Figure 5). It should be noted that these are guide values because not only NaOH was leached from geopolymer samples but also other substances that had an impact on conductivity. This method was used as an indicative method for determining the amount of substances leached from geopolymers.

Figure 5 shows the amount of NaOH calculated on the assumption that the measured conductivity values are solely due to the leaching of NaOH from the geopolymer. For calculations, it was assumed that the number of grams of NaOH in the tested sample solution is 4 times smaller than that resulting from the concentration corresponding to the measured conductivity, because the volume of the solution is 4 times smaller. The volume of solution is important because when pouring larger volumes of samples, the leaching should proceed faster due to the slower leveling of NaOH concentration in the sample and solution (more effective diffusion).

**Figure 6:**
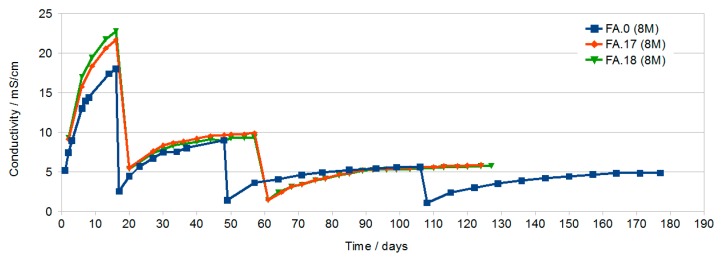
Conductivity of samples FA.0 (8 M), FA.17 (2% TiO_2_), and FA.18 (4% TiO_2_) as a function of immersion time with periodic water changes.

As shown in Figure 6, the conductivity of the solution for samples containing 2% and 4% TiO_2_ is greater than for samples without additives. This may be due to higher porosity or differences in material structure resulting from the effect of TiO_2_ on polycondensation.

**Figure 7:**
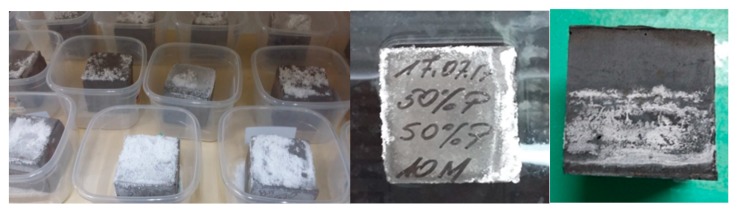
Appearance of selected samples during tests for susceptibility to efflorescence (after 14 days); samples cured at ambient conditions.

**Figure 8:**
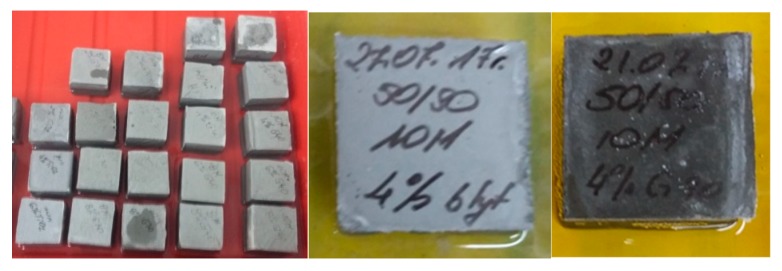
Appearance of selected samples during tests for tendency to efflorescence (after 60 days), samples cured in water.

The appearance of selected samples during tests for the tendency to efflorescence (after 14 days, cured at ambient conditions) is shown in Figure 7. The appearance of selected samples during tests for the tendency to efflorescence (after 60 days, cured in water) is shown in Figure 8.

Conductivity testing of the solutions in which the geopolymer samples were immersed showed a large increase in the conductivity of all solutions during the first dozen or so days of immersion. With the increase in time, a gradual decrease in the increase in conductivity as a function of time was found. The highest conductivity after about 16 days was found in solutions in which samples prepared with the use of activator at a concentration of 8 M and then 10 M were immersed. This may be due to the higher open porosity in these samples compared to samples containing G40.

After subsequent water changes, the differences in conductivity were less visible.

The final measurements made after about 76 days and two water changes showed similar conductivity of the solutions at the level of 5 mS/cm. In samples with rinsing solutions that have conductivity at this level, no efflorescence was later found (Figure 8).

### 3.2. Results of Compressive Strength

**Figure 9:**
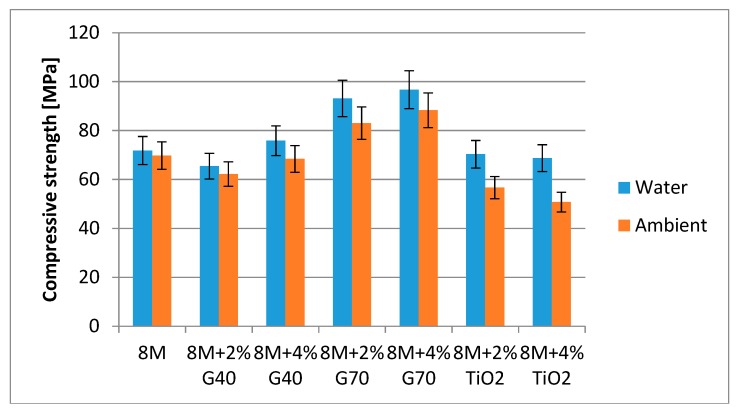
Results of geopolymer compressive strength after 60 days.

Figure 9 presents the results of compressive strength of the tested compositions. The tests were carried out after 60 days of curing in specified conditions.

Figure 9 presents the results of compressive strength of the tested compositions. The highest compression strength value is characteristic for samples with 4% G70 cement—almost 100 MPa. Samples without any additions had a compressive strength of 70 MPa. The addition of nanometric titanium oxide reduced the strength of geopolymer samples. It was also noted that all samples, both with and without additives, are characterized by a higher value of compressive strength after curing/maturing in water.

## 4. Conclusions

Studies carried out so far have shown that:

- The addition of 2% and 4% G70 significantly increases the compressive strength of geopolymers, both cured in ambient conditions and in water.
- The highest compressive strengths were obtained for geopolymers based on fly ash activated with 8 M NaOH solution and 4% G70 and cured in water—approx. 100 MPa.
- The addition of TiO_2_ reduced the compressive strength of geopolymer samples.
- Studies on the tendency to efflorescence have shown that special additives in the form of G40, G70, and TiO_2_ do not effectively protect geopolymers against efflorescence. The observations show that they only delay the formation time and the amount of arising efflorescence on the surface of the samples.
- Curing geopolymer samples in water conditions is an effective method of preventing efflorescence on their surfaces.
- Conductivity testing of solutions showed very low conductivity (approx. 0.5 mS/cm) for samples cured in water.

## Figures and Tables

**Table 1:** Oxide composition of fly ash.

	Oxide Composition (wt%)							
	SiO_2_	TiO_2_	Fe_2_O_3_	Al_2_O_3_	CaO	MgO	K_2_O	Na_2_O
Fly ash	55.9	1.09	5.92	23.49	2.72	2.61	3.55	0.59

**Table 2:** Contents of the main components in aluminum calcium cements introduced as additions to geopolymers.

Additions	Al_2_O_3_	CaO	SiO_2_	Fe_2_O_3_	Main Phase	Accompanying Phase
G40	>41%	>35.5%	<4%	<15%	CA	C_4_AF, C_12_A_7_, C_2_AS
G70	>69%	>28%	<0.5%	<0.3%	CA, CA_2_	C_12_A_7_, *α*A

**Table 3:** Composition of geopolymer composites with additives.

Description of Samples	Aqueous NaOH + Aqueous Sodium Silicate (Water Glass) (mL)	Fly Ash (g)	Sand (g)	Additives (g)
FA.0 (8 M)	120 (mL)+ 240 (mL)	1000	1000	−
FA.0 (10 M)	120 (mL) + 240 (mL)	1000	1000	−
FA.1 (8 M)	120 (mL) + 240 (mL)	1000	1000	20 g G40
FA.2 (8 M)	120 (mL) + 240 (mL)	1000	1000	40 g G40
FA.9 (8 M)	120 (mL) + 240 (mL)	1000	1000	20g G70
FA.10 (8 M)	120 (mL) + 240 (mL)	1000	1000	40g G70
FA.17 (8 M)	120 (mL) + 240 (mL)	1000	1000	20g TiO_2_
FA.18 (8 M)	120 (mL) + 240 (mL)	1000	1000	40g TiO_2_
